# Independent effects of exercise intensity on hepatic fat reduction in adults with metabolic dysfunction–associated steatotic liver disease: a randomized controlled trial protocol

**DOI:** 10.3389/fendo.2026.1711420

**Published:** 2026-02-10

**Authors:** Wei Huang, Yifei He, Rong Chen, Cuilan Huo, Yijun Xie, Yanyu Lin, Tian Wang, Xiangdi Dai, Junyu Wang, Weiqi Ruan, Haonan Zhai, Jie Zhuang, Jin Lu

**Affiliations:** 1 Department of Basic Education, Shanghai Customs University, Shanghai, China; 2 Department of Endocrinology, Changhai Hospital of Shanghai, Shanghai, China; 3 Shanghai University of Sport, School of Exercise and Health, Shanghai, China; 4 Faculty of Kinesiology, Sport and Recreation, University of Alberta, Edmonton, AB, Canada; 5 Fudan University Affiliated High School, Shanghai, China

**Keywords:** exercise intensity, hepatic fat content, high-intensity interval training, metabolic dysfuncIon–associated steatotic liver disease, moderate-intensity continuous training

## Abstract

**Importance:**

Exercise is considered a cornerstone of Metabolic dysfunction–associated steatotic liver disease (MASLD) management, but the independent role of exercise intensity in hepatic fat reduction has not been fully elucidated in clinical practice. The primary objective in this study is to evaluate the difference in exercise-induced reduction of hepatic fat content (HFC) between energy-matched high-intensity interval training (HIIT) and moderate-intensity continuous training (MICT). Furthermore, this study aims to investigate whether high-intensity exercise with lower exercise volume can yield comparable HFC reductions to moderate-intensity continuous training.

**Methods:**

This single-center, three-arm, open-label randomized controlled trial will recruit 105 adults aged 18–45 years with MASLD. Participants will be randomized in a 1:1:1 ratio to HIIT, MICT, or a low-volume HIIT (LHIIT). The sample size of 35 participants per group was calculated to provide 90% power to detect a significant difference in HFC reduction between the HIIT and MICT groups (two-sided α = 0.05, accounting for 20% dropout). The supervised exercise training will be conducted at a community fitness center for 12 weeks. The primary outcome is the absolute change in HFC, measured by magnetic resonance imaging–proton density fat fraction. Secondary outcomes include anthropometric parameters, abdominal adipose tissue, body composition, cardiometabolic biomarkers, and cardiorespiratory fitness. The analysis of the primary and secondary outcomes will be conducted according to the intention-to-treat principle.

**Discussion:**

This trial rigorously evaluates the independent effect of exercise intensity on HFC under energy-matched conditions, while simultaneously exploring the therapeutic efficiency of low-volume interventions. The findings of this study are expected to provide a theoretical and practical basis for precise exercise prescriptions for the MASLD population. The results will be disseminated through a peer-reviewed journal.

**Clinical Trial Registration:**

https://www.chictr.org.cn/showprojEN.html?proj=279678, identifier ChiCTR2500107821.

## 1 Introduction

Obesity is a major driver of metabolic disorders, with its progression significantly increasing the prevalence of complications. In China, metabolic-associated steatotic liver disease (MASLD) is the most common obesity-related complication and the leading chronic liver disease (1). It is estimated that the number of people with MASLD in China will reach 314 million by 2030 (2). Excessive visceral fat, particularly hepatic fat accumulation, serves as a stronger predictor for cardiovascular diseases (CVDs) than obesity alone (3). Moreover, hepatic fat content (HFC) is closely associated with the onset of type 2 diabetes (T2DM) (4). Therefore, early intervention for individuals with MASLD is essential to delay disease progression and reduce the risk of T2DM and CVDs.

Currently, lifestyle modifications, including dietary changes and increased physical activity, are the primary treatment approaches for MASLD (5). Among these, exercise has shown a dose-response relationship with reductions in HFC (6), and importantly, its effects may be independent of body weight changes (7). Specifically, high-intensity interval training(HIIT) demonstrates a similar reduction in HFC compared to moderate-intensity continuous training (8). Current guidelines propose that exercise volume, rather than intensity, is the principal determinant of HFC reduction (9). Conversely, Winn et al. reported that isoenergetic HIIT resulted in an 84% greater HFC reduction than MICT (10). However, this finding lacked statistical significance, which was attributed to the insufficient sample size (10). Moreover, Taylor et al. observed a non-significant trend (p=0.077) favoring HIIT over moderate-intensity continuous training (MICT) for hepatic fat reduction following a 3-month intervention (3 times/week) in cardiac rehabilitation individuals (11). Consequently, it remains uncertain whether, for a given exercise volume, higher intensity exercise offers an independent advantage over lower intensity exercise for mitigating liver fat accumulation. AMP-activated protein kinase (AMPK) serves as a pivotal signal modulator of exercise adaptation, functioning as an ‘energy switch’ that regulates cellular energy homeostasis (12). Emerging research indicates that AMPK and its downstream signaling pathways are closely linked to the mitigation of hepatic fat accumulation, positioning AMPK agonists as potential therapeutic targets for MASLD (13). As a cellular energy sensor, AMPK is primarily activated by elevations in ADP concentration, a metabolic perturbation that is significantly amplified by higher exercise intensities (14). From this perspective, short-duration high-intensity exercise holds significant therapeutic potential for reducing HFC in the MASLD population. Additionally, higher exercise intensity can achieve faster consumption of muscle glycogen (15), which helps to improve the competitive inhibition of substrate oxidation under the condition of high substrate flux after meals (13, 16, 17), thereby reducing the accumulation of fat content in the liver. We hypothesize that exercise-induced muscle glycogen depletion may be a regulatory pathway for exercise to reduce HFC, and thus higher exercise intensity may be more efficient in reducing HFC.

Therefore, the present study aims to clarify the independent role of exercise intensity in hepatic fat reduction among individuals with MASLD. To achieve this, we will primarily compare HIIT with MICT under matched energy expenditure. In addition, a low-volume HIIT group with approximately half the prescribed energy expenditure has been included to examine whether high-intensity exercise yields comparable efficiency in reducing HFC with high-volume moderate intensity exercise. We hypothesize that under energy-matched conditions, HIIT will elicit a significantly greater reduction in HFC compared to MICT, while low-volume HIIT will induce comparable reductions to MICT despite a lower total energy expenditure. This design will enable a comprehensive evaluation of exercise intensity as a critical determinant of exercise-induced changes in HFC.

## 2 Methods

### 2.1 Research design

**Figure 1:**
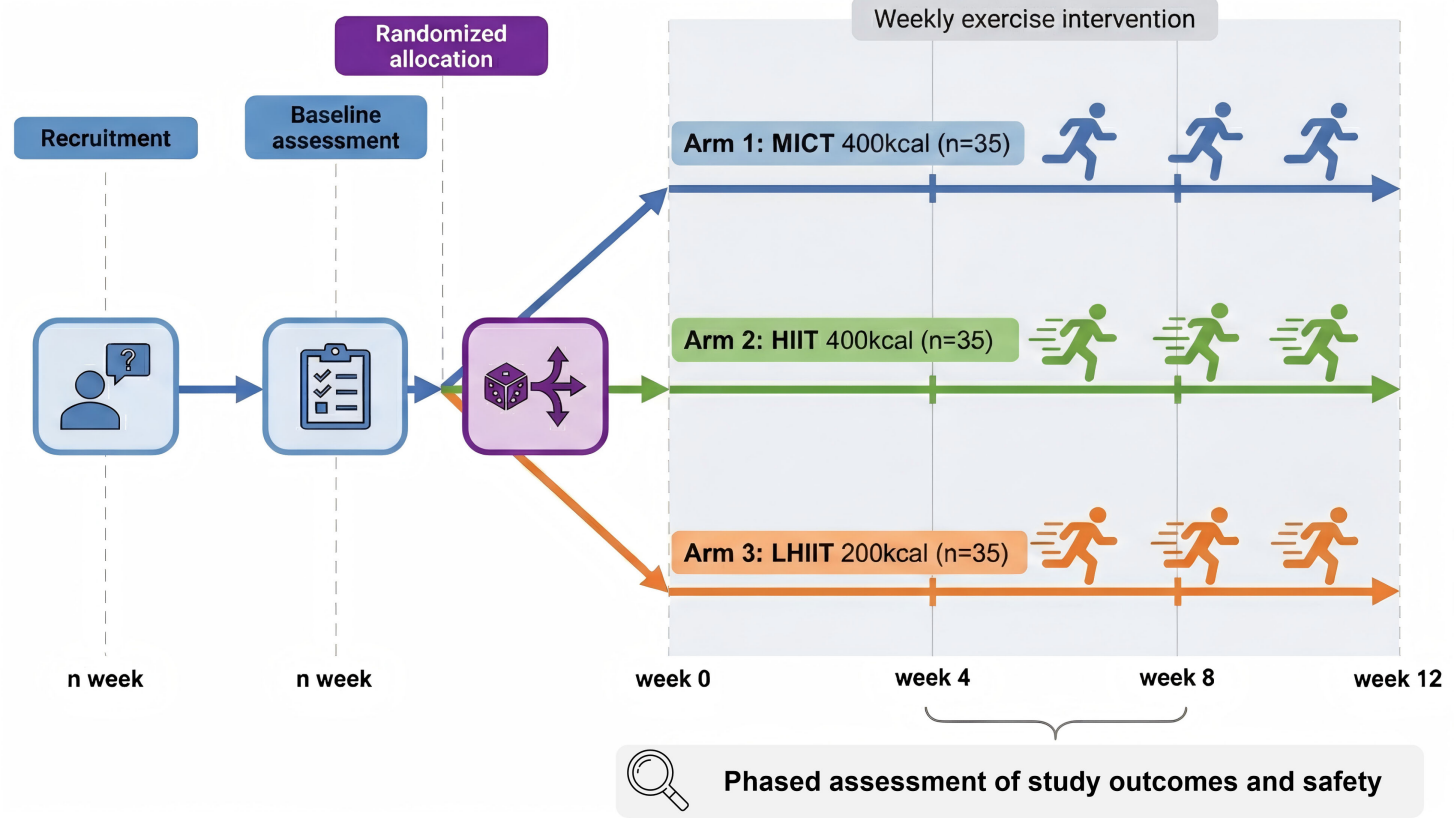
Flow paradigm.

This trial is a single-center, three-arm, open-label randomized controlled study (**Figure 1**). As a superiority trial, a non-exercise control group will not be included, given that numerous studies have already demonstrated the significant efficacy of aerobic training for decreasing HFC in individuals with MASLD. A total of 105 eligible participants aged 18–45 years will be randomized in a 1:1:1 ratio to the high-intensity interval training (HIIT) group, moderate-intensity continuous training (MICT) group, or low-volume HIIT (LHIIT) group. The intervention will last 12 weeks. The HIIT and MICT groups will perform exercise sessions aiming for approximately 400 kcal per session (~1200 kcal/week), while the LHIIT group will perform shorter sessions of approximately 200 kcal per session. To minimize confounding factors, all participants will receive standardized dietary guidance to maintain a stable caloric intake consistent with their baseline habits during the intervention period. All exercise sessions will be supervised by certified exercise trainers to ensure adherence, safety, and fidelity to the protocol. Due to the nature of the interventions, participants and trainers cannot be blinded; however, outcome assessors, imaging analysts, and statisticians will remain blinded to allocation. The primary outcome is the change in HFC, quantified by magnetic resonance imaging–proton density fat fraction (MRI-PDFF), with the primary comparison set between HIIT and MICT. Analyses involving the LHIIT group are considered exploratory and will be interpreted as hypothesis-generating. This trial protocol has been reviewed and approved by the Ethics Committee of the First Affiliated Hospital of Naval Medical University (CHEC2025-253) and registered on the Chinese Clinical Trial Registry (ChiCTR2500107821). Any modifications to the protocol will be fully documented and described in detail in subsequent publications.

### 2.2 Subjects

This study aims to recruit a total of 105 participants at the First Affiliated Hospital of Naval Medical University, with recruitment scheduled from January 2026 to January 2028. **Table 1** summarizes the detailed inclusion and exclusion criteria for participant selection. Recruitment will be facilitated through hepatology and weight management outpatient clinics at the hospital. Additionally, informational flyers will be distributed in community hospitals and health examination centers within a 5-kilometer radius of the main hospital to expand outreach efforts. Potential participants will be initially screened through review of their electronic medical records by study investigators to assess eligibility. Eligible individuals will then be invited to provide written informed consent by research staff, followed by completion of baseline assessments within four weeks of enrollment.

**Table 1:** Inclusion and exclusion criteria.

Inclusion criteria
- Age between 18 and 45 years, with no restrictions on gender
- Meets the diagnostic for MASLD
- Medication type and dosage have remained stable over the past three months
- No regular exercise routine(3 sessions/week, 30 minutes/session)
Exclusion criteria
- Weight change exceeding 5% in the past three months
- Inability to maintain blood glucose and blood pressure within normal ranges despite standard treatment
- Inability to participate in exercise testing and training due to any condition
- Women who are pregnant, planning to conceive, or in the periconceptional period
- Currently undergoing any form of hormone therapy.

**Table 1** outlines the inclusion and exclusion criteria. Individuals meeting the following criteria will be recruited: 1) An age range of 18 to 45 years, with no gender restrictions; 2) Fulfilling the diagnostic criteria for MASLD, as defined by Eslam et al. (18); 3) A stable medication type and dosage over the past three months; 4) No regular exercise routine (defined as participating in physical activity fewer than three times per week, with each session lasting less than 30 minutes, over the past three months). Individuals presenting with any of the following conditions will be excluded: 1) Weight change exceeding 5% in the past three months; 2) Inability to maintain blood glucose and blood pressure within normal ranges despite standard treatment; 3) Inability to participate in exercise testing and training due to any condition (e.g., severe cardiovascular, pulmonary, or renal diseases, musculoskeletal injuries, etc.); 4) Women who are pregnant, planning to conceive, or in the periconceptional period; 5) Currently undergoing any form of hormone therapy.

### 2.3 Randomization

A stratified block randomization method will be employed in this study. Participants will be randomly assigned to the following three groups: MICT group (n=35), HIIT group (n=35), and LHIIT group (n=35). Stratification is based on sex (male and female) and age. Stratification will be based on participant gender (Male, Female) and age group (18–30 years and 31–45 years), resulting in four distinct strata. Within each stratum, a computer-generated randomization sequence will be created using permuted blocks of size 6. The randomization list was generated prior to study commencement using R 4.5.1. To ensure allocation concealment, sequentially numbered, opaque, sealed envelopes will be prepared in advance based on the stratified randomization list. Following eligibility confirmation and informed consent, the research coordinator allocated the participant to the treatment group indicated in the next available envelope within the appropriate stratum.

### 2.4 Assessments and procedures

All study outcomes and corresponding assessment time points are presented in **Table 2**. The primary outcome is the change in HFC, as measured by magnetic resonance imaging, from baseline to 12 weeks post-intervention. Secondary outcomes encompassed: (1) anthropometric parameters; (2) body composition and abdominal fat; (3) cardiorespiratory fitness; (4) cardiometabolic biomarkers; and (5) Indicators of liver inflammation and fibrosis. Detailed measurement procedures are described below.

**Table 2:** The study schedule.

Study phase	Screening	0 week	4 week	8 week	12 week
Informed consent	✓	–	–	–	–
Demographics	✓	–	–	–	–
Medical history	✓	–	–	–	–
Lifestyle counseling	✓	✓	✓	✓	✓
Medications report	✓	✓	✓	✓	✓
Physical and diet assessment	✓	✓	✓	✓	✓
Anthropometric assessment	✓	✓	✓	✓	✓
Randomization	✓	–	–	–	–
MRI-PDFF	–	✓	–	–	✓
Cardiometabolic biomarkers	✓	✓	–	–	✓
Liver inflammatory biomarkers	✓	–	–	–	✓
Body composition	✓	✓	–	–	✓
Cardiorespiratory fitness	✓	✓	–	–	✓
Quality of life	✓	✓	–	–	✓
Adherence	✓	✓	✓	✓	✓
AEs	✓	✓	✓	✓	✓

MRI-PDFF, Magnetic Resonance Imaging - Proton Density Fat Fraction; AEs, adverse events.

#### 2.4.1 Primary outcomes

The primary outcome is the absolute change in HFC, quantified as MRI-PDFF, from baseline to 12 weeks. All MRI-PDFF measurements will be acquired using a standardized multi-echo gradient-echo sequence on the same 3.0T scanner (Magnetom Prisma, Siemens Healthineers, Erlangen, Germany), using an 18-channel body and a 32-channel spine phased-array coil, conducted by a single professional technician who is blinded to group allocation. A multi-echo 3D spoiled gradient-echo sequence (Volumetric Interpolated Breath-hold Examination-Based, VIBE) integrated within the Siemens LiverLab sequence will be performed under a single end-expiratory breath-hold. Typical acquisition parameters included: repetition time (TR)< 12 ms, six asymmetric echo times (TEs, e.g., first TE ~1.2ms, echo spacing ~1.2ms), flip angle ~3-6°, axial orientation, field of view covering the entire liver, and parallel imaging. PDFF maps will be automatically generated by LiverLab, which employs a multi-peak fat spectral model and corrects for T2-weight decay. The liver will be segmented using an artificial intelligence algorithm embedded in the assessment program. Segmentations with a goodness-of-fit exceeding 95% will be accepted after reviewing the images and reports.

#### 2.4.2 Secondary outcomes

##### 2.4.2.1 Clinical assessments

Height and weight will be measured using an electronic scale (DC-250, TANITA, Japan). Waist and hip circumferences will be assessed by trained nurses following standardized procedures, with participants standing in an upright position. Waist circumference will be measured at the midpoint between the lower margin of the last palpable rib and the top of the iliac crest, while hip circumference will be measured at the widest point over the buttocks. In addition, blood pressure will be measured according to the established clinical guidelines. Participants remained seated and at rest for a minimum of 5 minutes before assessment. Measurements will be obtained on the right upper arm using a validated automated sphygmomanometer with an appropriately sized cuff. Two successive readings will be taken at 1–2-minute intervals, and the mean value will be used for subsequent analyses.

##### 2.4.2.2 Body composition and abdominal fat

Body composition will be assessed via dual-energy X-ray absorptiometry, which enables precise quantification of fat mass and lean mass distribution using dual-energy X-ray absorptiometry (Lunar Prodigy, GE Healthcare, USA). Participants will be required to fast for ≥8 hours prior to testing and keep a standardized supine position on the scanning table with limbs immobilized using foam supports. The upper extremities are abducted at 15°from the torso while maintaining full leg extension, ensuring optimal anatomical alignment for accurate cross-sectional analysis.

Concurrently with hepatic fat assessment, abdominal adipose tissue will be also quantified. A T1-weighted VIBE Dixon sequence will be performed, centered at the L4-L5 intervertebral disc level under a single breath-hold. Acquisition parameters are repetition time (TR) 4.3ms, dual echo times (TE1/TE2) 1.29/2.52ms, slice thickness 3.0 mm, voxel size 1.5 × 1.5 × 3.0 mm³, and flip angle 7°.

Image analysis will be performed using OsiriX software (Pixmeo SARL, Bernex, Switzerland). Five axial slices centered at the L4-L5 midpoint will be reconstructed. Subcutaneous adipose tissue (SAT) and visceral adipose tissue (VAT) areas will be manually delineated using region of interest (ROI) tools, and the mean area (cm^2^) across the five slices will be calculated.

#### 2.4.3 Cardiometabolic indicators

After a 10–12 hour overnight fast, blood samples will be collected from participants by the clinical laboratory of Changhai Hospital following standardized protocols. A comprehensive panel of serum biochemical parameters will be assessed, including fasting glucose, insulin, glycated hemoglobin (HbA1c), total cholesterol (TC), triglycerides (TG), high-density lipoprotein cholesterol (HDL-C), low-density lipoprotein cholesterol (LDL-C). Insulin resistance will be evaluated using the homeostasis model assessment for insulin resistance (HOMA-IR) (19).

##### 2.4.3.1 Indicators of liver inflammation and fibrosis

We quantified a range of biomarkers associated with liver inflammation and fibrogenesis, including, alanine aminotransferase(ALT), and aspartate aminotransferase (AST), adiponectin, cytokeratin-18 (CK-18), fibroblast growth factor-21 (FGF-21), procollagen type III N-terminal propeptide (PIIINP), ferritin, and tissue inhibitor of metalloproteinases-1 (TIMP-1). The degree of hepatic fibrosis will be additionally estimated using validated non-invasive indices, specifically the Fibrosis-4 index (FIB-4) (20) and the AST-to-Platelet Ratio Index (APRI).

##### 2.4.3.2 Cardiorespiratory fitness

A treadmill-based maximal cardiopulmonary exercise test (CPET) will be performed in conjunction with indirect calorimetry to accurately measure peak oxygen uptake (VO_2peak_). After a 5-minute low-intensity warm-up and familiarization, participants underwent a graded exercise protocol with treadmill (h/p/cosmos sports & medical gmbh, Germany) and wearable metabolic system (Cosmed K5, Italy) using the modified Bruce protocol (21). The modified protocol incorporated two additional low-intensity stages at the beginning of the original protocol. This adaptation is specifically suitable for individuals with metabolic disorders. Therefore, the modified Bruce protocol enhanced test tolerability and safety while allowing for more accurate determination of VO_2peak_ in clinical populations with limited exercise capacity. During the incremental exercise test, participants’ exertion levels will be monitored using the Borg Rating of Perceived Exertion scale, for which standardized instructions will be provided prior to testing. Heart rate will be continuously recorded by heart rate monitor (Polar H10, Finland). Due to the compromised CRF commonly observed in this clinical population, a clear oxygen uptake plateau is often not achieved during the incremental exercise test. Therefore, the VO_2peak_ will be determined based on the attainment of at least two of the following criteria:1) Measured heart rate within 10 beats per minute of the age-predicted maximum (calculated as 207 - 0.7 * age in years); 2) Respiratory exchange ratio (RER) ≥ 1.10, and 3) Borg RPE score ≥ 18. Following the test, participants engaged in a cool-down period involving light walking or slow cycling until their heart rate returned to baseline levels. The testing will be conducted by a qualified exercise physiologist, and continuous access to medical support will be ensured throughout the procedure.

##### 2.4.3.3 Dietary and physical activity assessment

At baseline, participants will be required to complete a 3-day dietary record (two weekdays and one weekend day) using a mobile application (BOHE Corporation, Shanghai) to assess their habitual energy intake. Based on this baseline assessment, a dietitian will provide standardized dietary guidance to ensure caloric stability. During the intervention, participants will use the same mobile application to record their dietary intake on three randomly selected days (two weekdays and one weekend day) every two weeks. If the calculated average daily energy intake deviates from the baseline level by more than 10%, the dietitian will provide immediate feedback to facilitate a return to habitual intake levels.

The Global Physical Activity Questionnaire (GPAQ) will be used to investigate the physical activity level of participants. The GPAQ is particularly well-suited for assessing habitual physical activity patterns in adults. Its structured format aligns closely with the typical daily activity profiles of adult populations, making it an appropriate tool for this study.

### 2.5 Intervention

**Figure 2:**
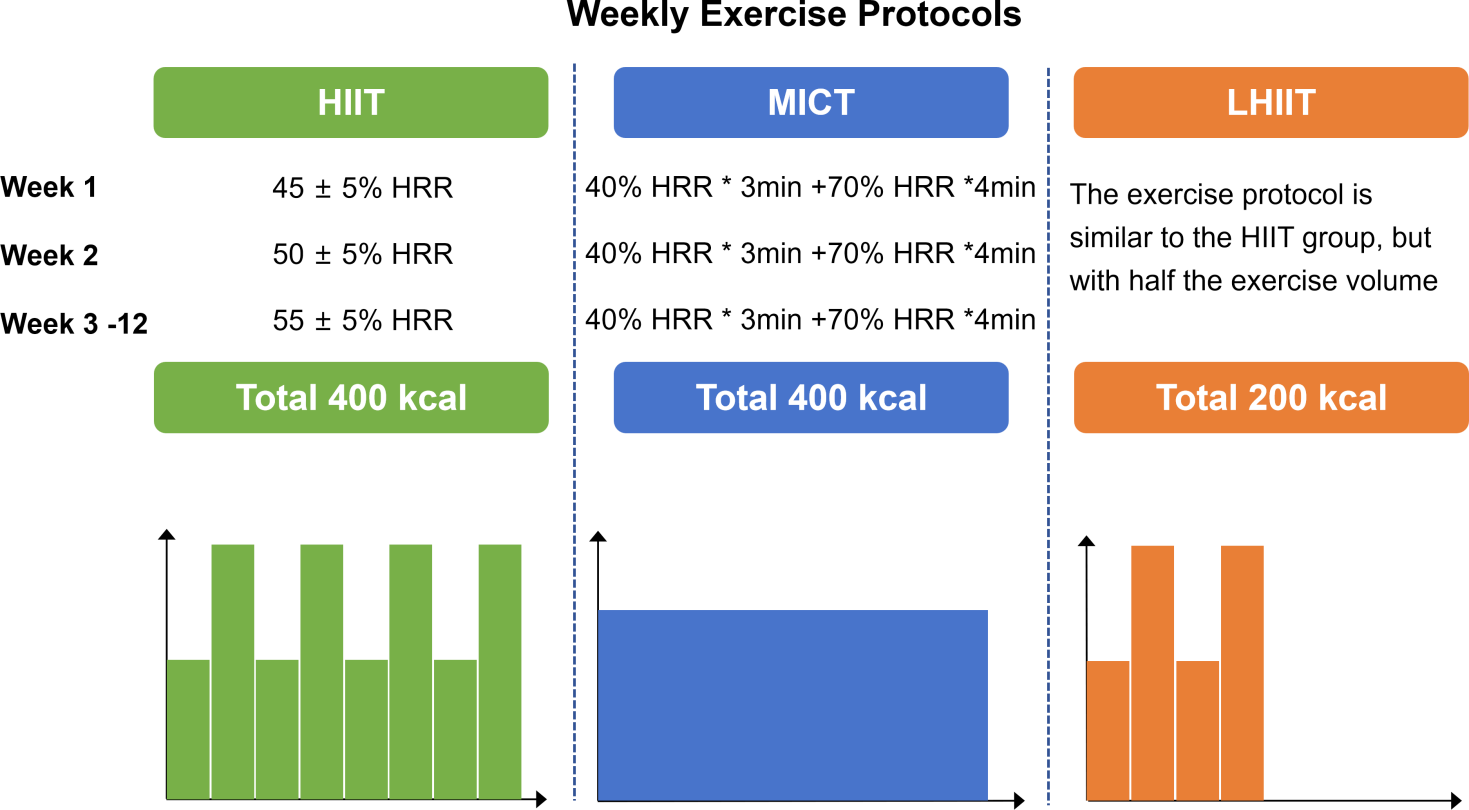
Exercise load. MICT, moderate intensity continuous training; HIIT, high-intensity interval training; LHIIT, low-volume high-intensity interval training; HRR, heart rate reserve.

The intervention period will last for 12 weeks, with 3 supervised training sessions per week at the community fitness center. Each session will consist of a warm-up (5–10 minutes), a main exercise phase (30–60 minutes) on a treadmill, and a cool-down (3–5 minutes). To maximize training adaptations while ensuring safety, we have designed a progressive intensity protocol. Specifically, the MICT group will exercise at 45–55% of Heart Rate Reserve (HRR). The HIIT and LHIIT groups will perform high-intensity intervals (4-minute work intervals at 70–90% HRR separated by 3-minute active recovery intervals at 40% HRR) and the training load will be adjusted monthly in accordance with the CPET. The detailed progression of intensity and heart rate targets is presented in **Figure 2**.

To ensure precise energy matching, individual energy expenditure will be calculated based on physiological data obtained from the baseline CPET. An individual linear regression equation establishing the relationship between HR and VO_2_ will be generated for each participant. Based on this relationship, the target energy expenditure is calculated using the caloric equivalent of oxygen (~5 kcal/L), and the session time will be calculated by following formula.


$$Exercise\;time=\;\frac{Targeted\;energy\;consumption\left(kcal\right)}{Exercise\;VO_{2}\left(L\cdot min^{-1}\right)*5\left(kcal\cdot L^{-1}\right)}$$

For the HIIT protocol, the total number of cycles is calculated based on the energy expenditure per cycle, using the following formula. In the final cycle, the duration of both high- and low-intensity intervals will be proportionally adjusted to ensure accurate achievement of the total energy expenditure goal.


$$N_{\left(cycles\right)}=\;\frac{Targeted\;energy\;consumption\left(kcal\right)}{\left[VO_{2high}*4+VO_{2low}*3\right]\left(L\cdot min^{-1}\right)*\;5\left(kcal\cdot L^{-1}\right)}$$

N, total number of cycles.

To account for improvements in cardiorespiratory fitness and ensure the maintenance of the prescribed relative intensity, CPET will be repeated at Week 4 and Week 8. The exercise prescription (target heart rates and session duration) will be recalibrated based on these updated physiological parameters. During all training sessions, participants’ heart rates will be monitored in real-time using the Polar OH1(Polar, Finland).

### 2.6 Sample size

The sample size is calculated based on the primary comparison between the HIIT and MICT groups regarding the absolute change in MRI-PDFF from baseline to 12 weeks. Based on the previous study that implemented a similar intervention duration, we anticipated an absolute reduction in hepatic fat content of -1.7% (SD 1.1%) for the HIIT group and -0.9% (SD 0.7%) for the MICT group (22). Assuming a pooled standard deviation of 0.92% and a two-sided significance level of 0.05, a sample size of 28 participants per group is required to achieve 90% statistical power to detect the between-group difference. To account for a potential dropout rate of 20%, the sample size is adjusted to 35 participants per group. Consequently, a total of 105 participants will be recruited, with equal allocation (1:1:1) to the HIIT, MICT, and LHIIT groups.

### 2.7 Statistical analysis plan

Baseline characteristics and study outcomes will be summarized using descriptive statistics. The normality of continuous variables will be assessed using the Shapiro-Wilk test and visual inspection of histograms and Q-Q plots. Continuous variables with a normal distribution will be presented as means ± standard deviations (SD), while non-normally distributed variables will be reported as medians and interquartile ranges (IQR). Treatment effects will be reported as the estimated mean difference (MD) between groups with 95% confidence intervals (CIs) to provide a clear estimation of the effect size. Categorical variables will be expressed as frequencies and percentages (n, %). Baseline differences between groups will be assessed using one-way ANOVA or Kruskal-Wallis tests for continuous variables, and Chi-square tests or Fisher’s exact tests for categorical variables.

The analysis will be conducted according to the Intention-to-Treat (ITT) principle, including all randomized participants (N = 105) for both primary and secondary endpoints. To address missing data arising from participant withdrawal, Multiple Imputation by Chained Equations (MICE) (m=20 imputed datasets) will be performed prior to the primary analysis. Treatment effects will be evaluated using analysis of covariance (ANCOVA). The post-intervention measurements will be included as the dependent variable. Fixed effects will include group (HIIT, MICT, LHIIT), and the baseline measurements will be included as a covariate. The results from the imputed datasets will be pooled using Rubin’s rules. Tukey’s Honestly Significant Difference test will be used for *post-hoc* pairwise comparisons. Furthermore, sensitivity analyses for primary outcome will be conducted to assess the robustness of the results. These will include: (1) a Per-Protocol analysis; (2) an analysis excluding participants with >5% body weight change; and (3) an analysis including percentage body weight change as a covariate in the primary ANCOVA model. All statistical analyses will be performed using R software (version 4.5.1). A two-sided p-value< 0.05 will be considered statistically significant.

### 2.8 Compliance and adherence

At enrollment, all participants will receive standardized education to ensure full understanding of the intervention requirements and the importance of adherence. Exercise sessions will be delivered under direct supervision by certified exercise trainers, with adherence monitored through detailed attendance records and continuous heart rate tracking. To enhance participant adherence and motivation, participants will receive personalized feedback on their progress from the researchers, and will be contacted via text message or phone calls if they miss more than one session. Furthermore, to maintain consistency in habitual dietary intake, participants will be required to complete a 3-day dietary record (two weekdays and one weekend day) using a mobile application every two weeks. These logs, including food logs and meal photographs, will be reviewed by a study dietitian, who will provide immediate feedback if average daily energy intake deviates by more than $10\%$ from the established baseline level.

Adherence will be quantified as the proportion of prescribed sessions attended and completed within the target training intensity zone. The minimum adherence threshold for inclusion in the per-protocol analysis is set at 70%. Participants whose adherence falls below this level will receive additional counseling and behavioral support from the research team. If adherence remains inadequate despite these measures, withdrawal from the trial may be considered. All cases of withdrawal and their reasons will be documented and transparently reported in the final trial outcomes.

### 2.9 Safety

This trial will be conducted in accordance with the Declaration of Helsinki and established clinical research guidelines. Prior to enrollment, all participants will provide written informed consent after being fully informed of the potential risks and benefits of participation. Baseline health assessments will be performed by qualified physicians to confirm eligibility and ensure participants’ suitability for exercise testing and interventions. Throughout the intervention period, participants’ health status will be closely monitored, with systematic documentation of any safety-related information, including modifications to the exercise regimen, participant-reported symptoms, and monitoring results. Additionally, the trial will be immediately discontinued for any participant presenting with: (a) SBP > 180 mmHg or DBP > 110 mmHg; (b) exercise-induced angina or dyspnea; (c) syncope or dizziness; or (d) severe musculoskeletal injury. Any affected participant will receive appropriate medical treatment. Emergency medical support will be available on site during all exercise sessions.

### 2.10 Data management

Study data will first be documented on standardized paper case report forms and then independently entered into a secure electronic database by two trained research staff members to ensure accuracy. The database is protected with role-based access controls, allowing only authorized personnel to view or modify study data. Routine quality assurance procedures will be performed, including verification of consistency between original records and electronic entries, checks for data completeness and logical integrity, and identification of outliers or missing values. Any discrepancies will be promptly reviewed and resolved in consultation with the principal investigator. In compliance with regulatory requirements and the approval of the institutional ethics committee, all study-related materials, including raw data, processed datasets, analysis outputs, and biological samples, will be securely archived for a minimum of five years following study completion.

Regarding data sharing, in line with principles of transparency and reproducibility, de-identified study data may be made available to qualified researchers upon reasonable request. Access to the dataset will require prior approval from the principal investigator and agreement to use the data solely for scientific purposes consistent with the aims of the study. Requests for data access will be documented, and approved users will be required to comply with institutional and ethical guidelines for secondary data use.

An independent Data Monitoring Committee (DMC) will not be established for this trial. The study is a single-center, investigator-initiated clinical trial involving exercise interventions of moderate intensity, which are generally considered low to moderate risk. Given the relatively small sample size, the short intervention period (12 weeks), and the non-pharmacological nature of the intervention, the establishment of an independent DMC is not deemed necessary.

### 2.11 Adverse events

All AEs will be documented in detail, including their type, timing, severity, management strategies, and clinical outcomes. Particular attention will be paid to exercise-related musculoskeletal injuries, cardiovascular incidents, and symptoms of overtraining. Investigators will receive pre-trial training to ensure standardized identification, documentation, and reporting of AEs.

SAEs, irrespective of their potential association with the study intervention, will be reported in accordance with regulatory requirements. Each SAE will be submitted in writing to the Ethics Committee within seven calendar days of occurrence or identification. Non-serious adverse events will also be documented and subjected to periodic review to ensure participant safety and data integrity. Safety data will be reviewed regularly by the study team and Ethics Committee.

## 3 Discussion

MASLD is the most prevalent chronic liver disease worldwide (23), strongly linked to increased cardiometabolic risk and underscoring the critical role of exercise interventions in its management (9, 24). This study was specifically designed to examine the effect of exercise intensity on HFC in individuals with MASLD. By employing MRI-PDFF as a standardized and reproducible outcome measure and balancing energy consumption between groups, the study design enhances methodological rigor. The anticipated findings may provide clinically relevant insights that contribute to refining exercise prescriptions for MASLD, offering more precise recommendations regarding exercise intensity while aligning with current international guidelines.

This study is a three-arm parallel randomized controlled trial and, to the best of our knowledge, represents the first attempt to investigate the effects of exercise intensity on HFC reduction under both energy-matched and unmatched conditions. Previous studies attempting to isolate the effect of exercise intensity on HFC in patients with NAFLD have been limited by methodological constraints. For instance, Zhang et al. conducted a long-term intervention (12 months); however, the differences in total exercise volume, compounded by the reduction in intensity within the high-intensity group during the intervention, obscured the specific role of intensity (25). Similarly, Keating et al. investigated the impact of exercise dose on HFC reduction across multiple dimensions(frequency, intensity, and volume); however, the simultaneous manipulation of these variables introduced excessive confounding factors, precluding a clear delineation of the intensity-specific effects (26). To address the volume confounder, Winn et al. implemented an energy-matched design comparing HIIT and MICT. Although they reported a clinically relevant trend where HIIT reduced HFC by 84% more than MICT, the study was underpowered due to a small sample size and short duration (4 weeks), resulting in a lack of statistical significance (10). Filling the gap for lower-volume protocols, Sabag et al. found that 12 weeks of low-volume HIIT reduced HFC by 89% more than MICT, but no statistical difference was observed between groups (22). These findings suggest a potential superiority of higher intensity, but the evidence remains inconclusive. As highlighted in a meta-analysis by Sabag et al., there is a paucity of studies directly comparing HIIT and MICT, with Winn et al. being the only prior study to strictly match energy expenditure. Therefore, our study is critical to filling this void by rigorously investigating the independent role of exercise intensity under both matched and unmatched energy conditions.

Previous studies have consistently suggested that exercise volume is a major determinant of hepatic fat reduction in individuals with MASLD (6, 9, 24). However, emerging evidence indicates that the beneficial effects of exercise on hepatic steatosis cannot be fully explained by volume or energy deficit alone, highlighting the potential importance of exercise intensity as an independent factor (7, 27, 28). Impey et al. proposed the “muscle glycogen threshold hypothesis,” which suggests that when skeletal muscle glycogen is reduced to a critical level—through diet or exercise—it triggers greater metabolic adaptations, including enhanced fatty acid oxidation (15). Because higher-intensity exercise accelerates glycogen depletion (29), it may induce these adaptations more efficiently, thereby amplifying metabolic benefits. Furthermore, reduced glycogen availability may facilitate postprandial non-oxidative glucose disposal, a key element in improving metabolic flexibility (16). These mechanistic insights provide a plausible explanation for why the effect of exercise-induced HFC reduction is partially independent of weight loss (7, 27, 30). Nevertheless, research directly comparing the differential effects of exercise modalities on hepatic fat reduction remains limited (8). The only trial that adequately controlled energy expenditure between moderate- and high-intensity exercise was underpowered due to a small sample size, making it difficult to draw firm conclusions regarding their relative efficacy (10).

It is crucial to clarify that our study does not negate the established impact of exercise volume on hepatic fat reduction. AMP-activated protein kinase, a widely accepted therapeutic target for exercise in MASLD, functions through a complex cascade integrating energy expenditure signals, upstream kinases, and downstream metabolic pathways (12). Consequently, its activation is influenced not only by short-duration high intensity but also by total exercise volume, the effect of which will be investigated by the AMPED study (31).

The distinct advantage of high-intensity intervention lies in its potential to achieve HFC reductions comparable to MICT with reduced time and energy expenditure, or potentially superior reductions when energy is matched. This provides diverse, efficient intervention options for the heterogeneous MASLD population. However, the optimal physiological boundaries for intensity and volume in this population remain undefined. It cannot be assumed *a priori* that the high-volume HIIT (400 kcal) will inherently outperform low-volume LHIIT (200 kcal). Individuals with metabolic dysfunction may exhibit impaired metabolic flexibility or maladaptation to high physiological loads (32, 33). Therefore, determining the effective physiological threshold and establishing safe dosage boundaries based on individual metabolic characteristics represent critical questions worthy of further investigation.

Collectively, the results of this study facilitate a flexible approach to exercise prescription for MASLD, tailoring the regimen to the time availability and fitness level of MASLD population. We propose that low-volume HIIT is an optimal strategy for time-poor individuals, while high-volume HIIT serves those prioritizing maximal fat reduction. For patients unable to tolerate high intensities, volume-compensated moderate-intensity exercise offers a dependable alternative for managing HFC.

## 4 Potential limitation

First, as with most exercise interventions, it is not possible to blind participants or exercise trainers to the group allocation. This could theoretically introduce performance bias. However, to mitigate this risk, outcome assessors (specifically the MRI technicians and analysts) and statisticians will remain blinded to group allocation, and the primary outcome is an objective quantitative measure less susceptible to subjective bias.

Second, while all participants will receive standardized dietary guidance to maintain caloric stability, strictly controlled feeding (e.g., providing all meals) is not employed. Although this may introduce some variability in energy intake, this design was chosen to enhance the ecological validity of the study, reflecting a real-world clinical setting where patients manage their own diet. To mitigate this, we have established a rigorous monitoring system: participants’ dietary intake will be tracked weekly via a mobile app, and immediate feedback will be provided by a dietitian if energy intake deviates by more than 10% from baseline. Furthermore, body weight change will be included as a covariate in the sensitivity analysis to isolate the independent effect of exercise intensity.

Third, the study is conducted at a single center with a specific population. While this ensures high consistency in intervention delivery and data collection, it may limit the generalizability of the findings to other ethnic groups or older populations. Future multi-center studies will be needed to validate these findings across broader demographics.

## References

[1] Chen K Shen Z Gu W Lyu Z Qi X Mu Y . Prevalence of obesity and associated complications in China: A cross-sectional, real-world study in 15.8 million adults. Diabetes Obes Metab. (2023) 25:3390–9. doi: 10.1111/dom.15238, PMID: 37589256

[2] Estes C Anstee QM Arias-Loste MT Bantel H Bellentani S Caballeria J . Modeling NAFLD disease burden in China, France, Germany, Italy, Japan, Spain, United Kingdom, and United States for the period 2016–2030. J Hepatol. (2018) 69:896–904. doi: 10.1016/j.jhep.2018.05.036, PMID: 29886156

[3] Targher G Byrne CD Tilg H . NAFLD and increased risk of cardiovascular disease: clinical associations, pathophysiological mechanisms and pharmacological implications. Gut. (2020) 69:1691–705. doi: 10.1136/gutjnl-2020-320622, PMID: 32321858

[4] Stefan N Cusi K . A global view of the interplay between non-alcoholic fatty liver disease and diabetes. Lancet Diabetes Endocrinol. (2022) 10:284–96. doi: 10.1016/S2213-8587(22)00003-1, PMID: 35183303

[5] Ivancovsky Wajcman D Byrne CJ Dillon JF Brennan PN Villota-Rivas M Younossi ZM . A narrative review of lifestyle management guidelines for metabolic dysfunction–associated steatotic liver disease. Hepatology. (2024). doi: 10.1097/HEP.0000000000001058, PMID: 39167567 PMC13089823

[6] Smart NA King N McFarlane JR Graham PL Dieberg G . Effect of exercise training on liver function in adults who are overweight or exhibit fatty liver disease: a systematic review and meta-analysis. Br J Sports Med. (2018) 52:834–43. doi: 10.1136/bjsports-2016-096197, PMID: 27317790 PMC6029644

[7] Stine JG DiJoseph K Pattison Z Harrington A Chinchilli VM Schmitz KH . Exercise training is associated with treatment response in liver fat content by magnetic resonance imaging independent of clinically significant body weight loss in patients with nonalcoholic fatty liver disease: A systematic review and meta-analysis. Am J Gastroenterol. (2023) 118:1204–13. doi: 10.14309/ajg.0000000000002098, PMID: 36705333 PMC10287833

[8] Sabag A Barr L Armour M Armstrong A Baker CJ Twigg SM . The effect of high-intensity interval training vs moderate-intensity continuous training on liver fat: A systematic review and meta-analysis. J Clin Endocrinol Metab. (2022) 107:862–81. doi: 10.1210/clinem/dgab795, PMID: 34724062

[9] Keating SE Sabag A Hallsworth K Hickman IJ Macdonald GA Stine JG . Exercise in the management of metabolic-associated fatty liver disease (MAFLD) in adults: A position statement from exercise and sport science Australia. Sports Med. (2023) 53:2347–71. doi: 10.1007/s40279-023-01918-w, PMID: 37695493 PMC10687186

[10] Winn NC Liu Y Rector RS Parks EJ Ibdah JA Kanaley JA . Energy-matched moderate and high intensity exercise training improves nonalcoholic fatty liver disease risk independent of changes in body mass or abdominal adiposity — A randomized trial. Metabolism. (2018) 78:128–40. doi: 10.1016/j.metabol.2017.08.012, PMID: 28941598

[11] Taylor JL Holland DJ Mielke GI Bailey TG Johnson NA Leveritt MD . Effect of high-intensity interval training on visceral and liver fat in cardiac rehabilitation: A randomized controlled trial. Obes Silver Spring Md. (2020) 28:1245–53. doi: 10.1002/oby.22833, PMID: 32475048

[12] Spaulding HR Yan Z . AMPK and the adaptation to exercise. Annu Rev Physiol. (2022) 84:209–27. doi: 10.1146/annurev-physiol-060721-095517, PMID: 35143330 PMC8919726

[13] Bekheit M Kamera B Colacino L Dropmann A Delibegovic M Almadhoob F . Mechanisms underpinning the effect of exercise on the non-alcoholic fatty liver disease: review. EXCLI J. (2025). doi: 10.17179/EXCLI2024-7718, PMID: 40071029 PMC11895063

[14] Oakhill JS Scott JW Kemp Bruce E . AMPK functions as an adenylate charge-regulated protein kinase. Trends Endocrinol Metab. (2012) 23:125–32. doi: 10.1016/j.tem.2011.12.006, PMID: 22284532

[15] Impey SG Hearris MA Hammond KM Bartlett JD Louis J Close GL . Fuel for the work required: A theoretical framework for carbohydrate periodization and the glycogen threshold hypothesis. Sports Med. (2018) 48:1031–48. doi: 10.1007/s40279-018-0867-7, PMID: 29453741 PMC5889771

[16] Galgani J Ravussin E . Energy metabolism, fuel selection and body weight regulation. Int J Obes. (2008) 32:S109–19. doi: 10.1038/ijo.2008.246, PMID: 19136979 PMC2897177

[17] Galgani JE Heilbronn LK Azuma K Kelley DE Albu JB Pi-Sunyer X . Metabolic flexibility in response to glucose is not impaired in people with type 2 diabetes after controlling for glucose disposal rate. Diabetes. (2008) 57:841–5. doi: 10.2337/db08-0043, PMID: 18285553 PMC2756651

[18] Eslam M Newsome PN Sarin SK Anstee QM Targher G Romero-Gomez M . A new definition for metabolic dysfunction-associated fatty liver disease: An international expert consensus statement. J Hepatol. (2020) 73:202–9. doi: 10.1016/j.jhep.2020.03.039, PMID: 32278004

[19] Matthews DR Hosker JP Rudenski AS Naylor BA Treacher DF Turner RC . Homeostasis model assessment: insulin resistance and β-cell function from fasting plasma glucose and insulin concentrations in man. Diabetologia. (1985) 28:412–9. doi: 10.1007/BF00280883, PMID: 3899825

[20] Shah AG Lydecker A Murray K Tetri BN Contos MJ Sanyal AJ . Comparison of noninvasive markers of fibrosis in patients with nonalcoholic fatty liver disease. Clin Gastroenterol Hepatol Off Clin Pract J Am Gastroenterol Assoc. (2009) 7:1104–12. doi: 10.1016/j.cgh.2009.05.033, PMID: 19523535 PMC3079239

[21] Will PM Walter JD . Exercise testing: Improving performance with a ramped Bruce protocol. Am Heart J. (1999) 138:1033–7. doi: 10.1016/S0002-8703(99)70067-0, PMID: 10577432

[22] Sabag A Way KL Sultana RN Keating SE Gerofi JA Chuter VH . The effect of a novel low-volume aerobic exercise intervention on liver fat in type 2 diabetes: A randomized controlled trial. Diabetes Care. (2020) 43:2371–8. doi: 10.2337/dc19-2523, PMID: 32732374

[23] Teng ML Ng CH Huang DQ Chan KE Tan DJ Lim WH . Global incidence and prevalence of nonalcoholic fatty liver disease. Clin Mol Hepatol. (2023) 29:S32–42. doi: 10.3350/cmh.2022.0365, PMID: 36517002 PMC10029957

[24] Tacke F Horn P Wai-Sun Wong V Ratziu V Bugianesi E Francque S . EASL–EASD–EASO Clinical Practice Guidelines on the management of metabolic dysfunction-associated steatotic liver disease (MASLD). J Hepatol. (2024) 81:492–542. doi: 10.1016/j.jhep.2024.04.031, PMID: 38851997

[25] Zhang H-J He J Pan L-L Ma Z-M Han C-K Chen C-S . Effects of moderate and vigorous exercise on nonalcoholic fatty liver disease: A randomized clinical trial. JAMA Intern Med. (2016) 176:1074. doi: 10.1001/jamainternmed.2016.3202, PMID: 27379904

[26] Keating SE Hackett DA Parker HM O’Connor HT Gerofi JA Sainsbury A . Effect of aerobic exercise training dose on liver fat and visceral adiposity. J Hepatol. (2015) 63:174–82. doi: 10.1016/j.jhep.2015.02.022, PMID: 25863524

[27] Johnson NA Sachinwalla T Walton DW Smith K Armstrong A Thompson MW . Aerobic exercise training reduces hepatic and visceral lipids in obese individuals without weight loss. Hepatol Baltim Md. (2009) 50:1105–12. doi: 10.1002/hep.23129, PMID: 19637289

[28] Sargeant JA Gray LJ Bodicoat DH Willis SA Stensel DJ Nimmo MA . The effect of exercise training on intrahepatic triglyceride and hepatic insulin sensitivity: a systematic review and meta-analysis. Obes Rev. (2018) 19:1446–59. doi: 10.1111/obr.12719, PMID: 30092609

[29] Areta JL Hopkins WG . Skeletal muscle glycogen content at rest and during endurance exercise in humans: A meta-analysis. Sports Med. (2018) 48:2091–102. doi: 10.1007/s40279-018-0941-1, PMID: 29923148

[30] Houghton D Thoma C Hallsworth K Cassidy S Hardy T Burt AD . Exercise reduces liver lipids and visceral adiposity in patients with nonalcoholic steatohepatitis in a randomized controlled trial. Clin Gastroenterol Hepatol. (2017) 15:96–102.e3. doi: 10.1016/j.cgh.2016.07.031, PMID: 27521509 PMC5196006

[31] Stine JG Hummer B Smith N Tressler H Heinle JW VanKirk K . AMPED study: Protocol for a randomized controlled trial of different doses of aerobic exercise training. Hepatol Commun. (2024) 8:e0464. doi: 10.1097/HC9.0000000000000464, PMID: 38896071 PMC11186820

[32] Hammad S Ogris C Othman A Erdoesi P Schmidt-Heck W Biermayer I . Tolerance of repeated toxic injuries of murine livers is associated with steatosis and inflammation. Cell Death Dis. (2023) 14:414. doi: 10.1038/s41419-023-05855-4, PMID: 37438332 PMC10338629

[33] Malin SK Viskochil R Oliver C Braun B . Mild fasting hyperglycemia shifts fuel reliance toward fat during exercise in adults with impaired glucose tolerance. J Appl Physiol. (2013) 115:78–83. doi: 10.1152/japplphysiol.00084.2013, PMID: 23599396 PMC3727012

